# Effects of gestational diabetes mellitus on the quality and quantity of blood hematopoietic stem cells: a case-control study

**DOI:** 10.3325/cmj.2021.62.590

**Published:** 2021-12

**Authors:** Maiza Tusimin, Sara M. El. Ahmed, Kai Wei Lee, Ching Siew Mooi, Sabariah Md Noor, Norshariza Nordin

**Affiliations:** 1 Department of Obstetrics and Gynecology, Universiti Putra Malaysia, Serdang, Malaysia; 2 Department of Pathology, Universiti Putra Malaysia, Serdang, Malaysia; 3 Department of Pre-Clinical Sciences, Universiti Tunku Abdul Rahman, Kampar, Malaysia; 4 Department of Family Medicine, Universiti Putra Malaysia, Serdang, Malaysia; 5 Department of Biomedicine, Universiti Putra Malaysia, Serdang, Malaysia

## Abstract

**Aim:**

To evaluate the effects of gestational diabetes mellitus (GDM) on the quantity and quality of hematopoietic stem cells (HSC).

**Methods:**

In this case-control study, HSC were isolated from umbilical cord blood (UCB) procured at delivery from 63 mothers with GDM and 67 healthy mothers. Total nucleated cells (TNC) and CD34^+^ cells were quantified using BD FACSCalibur flow cytometer. The quantity and quality of stem cells were determined.

**Results:**

The GDM group had lower total cord blood volume and lower number of nucleated HSC compared with healthy mothers. Regarding stem cell quantity parameters, they had significantly lower UCB volume (*P* = 0.041), TNC count (*P* = 0.022), total viable NC count (*P* = 0.014), and CD34+ percentage (*P* = 0.014). Regarding the quality of stem cells, they had significantly lower viable TNC percentage (*P* = 0.015). The predictors for total TNC count were longer labor duration (adjusted B coefficient [p]: 0.031 [0.046]), greater estimated blood loss (0.089 [0.005]), female neonates (12.322 [0.049]), and higher placenta weight (0.080 [0.033]). The predictors of total viable NC count were greater estimated blood loss (0.092 [0.003]), female neonates (13.16 [0.035]), and greater placenta weight (0.083 [0.026]).

**Conclusion:**

The GDM group had much lower quantity and quality of UCB stem cells. Our results should be taken into consideration when drawing cord blood for unrelated stem cell banking in an obstetric unit to ensure the obtaining of optimal cord blood samples and to avoid unnecessary expenses.

Umbilical cord blood (UCB) is increasingly being used as a primitive source of hematopoietic stem cells (HSC). This has created the need for growing storage inventories, which contain a large number of genetically diverse UCB units available when there is no adult peripheral blood stem cell or bone marrow donor. Since the clinical outcomes of CB transplantation are affected by nucleated cell count per UCB unit transplanted, UCB units containing an appropriate number of nucleated cells must be obtained (1). However, the use of UCB entails several limitations compared with the use of other stem cell sources, including insufficient cell doses for larger recipients, delayed neutrophil and platelet engraftment, prolonged immune reconstitution, and lack of donor white blood cells (WBC) for donor WBC infusion (2-4).

The two main factors for the selection of blood cord units for cryopreservation are a minimum product weight (volume) of between 40 and 60 mL (5,6) and a total nucleated cell (TNC) count from 6 to 10 × 10^8^ for storage (7). The concentration of CD34^+^ cells may also affect engraftment and survival after UCB transplantation (8).

To date, most research has focused on the variables that can improve the quality of UCB since their greater understanding could reduce the cost and time required for evaluating, processing, and storing the material (9,10). UCB quality is affected by several maternal and fetal characteristics. Most studies investigated UCB stem cells in a healthy pregnancy. Data about the effects of common diseases complicating pregnancy are still scarce. Specifically, there is little data on the effects of gestational diabetes mellitus (GDM) on HSC isolated from UCB. The quantity of hematopoietic stem and progenitor cells (CD34^+^) in the UCB of neonates born to women with GDM on insulin therapy was much higher than that of neonates born to healthy women (11). Therefore, we assessed the quality and quantity of HSC isolated from UCB of neonates born to GDM mothers.

## PATIENTS AND METHODS

### Setting

This case-control study was conducted in the labor rooms at Hospital Serdang between May 2016 and April 2017. Hospital Serdang, located in Selangor State, is one of the centers involved in cord blood collection by the National Blood Bank of Malaysia. The study was approved by the Medical Research and Ethics Committee, Ministry of Health Malaysia (NMRR-14-1818-19019).

### Procurement of human umbilical cord

The cord was clamped in the usual way after the delivery of the baby but before placenta delivery. The umbilical cord blood was collected from the cut of the cord by trained professional midwives. Before UCB collection, the umbilical cord at the puncture site was disinfected.

### Study population

The inclusion criterion for cases was GDM diagnosed according to the WHO 2006 criteria (12) requiring only dietary modification, ie, 75-g oral glucose tolerance test 2-hour plasma glucose >7.8 mmol/L (140-199 mg/dL) and fasting glucose <7.0 mmol/L, at booking or at 24-28 weeks of gestation. The inclusion criterion for controls was uncomplicated normal pregnancy in healthy mothers. The general exclusion criteria were testing positive for infectious diseases (hepatitis B or C, human immunodeficiency virus, cytomegalovirus, or syphilis), any disorders not limited to hematological disorders, genetic disorders, vascular disorder (eg, preeclampsia and chronic hypertension), autoimmune diseases, kidney or liver disorders, multigravidity and carrying a fetus with congenital malformation detected by ultrasound examination or with congenital infection identified during the antenatal follow up.

### Sample size calculation

The sample size was calculated using the formula for case-control studies or for comparison between two groups when the endpoint is quantitative data. Based on Qiu et al (13), TNC count of 12.6 × 10^7^ /unit in the cord blood differentiates babies of mothers with preeclampsia from healthy neonates (13). Hence, we hypothesized that mean TNC of 12.6 would significantly differentiate newborns born to mothers with GDM from those born to healthy mothers. The estimated sample size was 50 for the GDM group and for the healthy group, with 80% power, a 95% confidence interval (CI), and the significance level of 5%. After considering the non-response rate of 20%, the total number of respondents needed in GDM and healthy group was 63.

### Cord blood collection

UCB was drawn by gravity into a 250-mL sterile bag collection set (All Eights (M) SDN BHD, Subang Jaya, Malaysia) containing 35 mL citrate phosphate-dextrose anticoagulant. The CB samples were labeled, stored on wet ice, and transported to the laboratory for processing. UCB collections are usually kept for not more than 24-48 hours at 4-8 °C before processing.

### Laboratory processing

The samples were stained and processed with the BD stem cell Enumeration Kit (BD Biosciences, Kuala Lumpur, Malaysia). CD34^+^ and TNC were counted by using flow cytometry following the International Society of Hematotherapy and Graft Engineering (ISHAGE) protocol. Briefly, reverse pipetting technique was used to add 100 μL of each UCB unit to BD Trucount tubes (BD Biosciences); UCB was then stained with 20 μL BD Stem Cell reagent and 20 μL 7-AAD reagent. After staining, erythrocytes were lysed by using ammonium chloride lysis solution, and the analysis was carried out within one hour with BD FACSCalibur flow cytometry.

### Biologic studies

The total collected UCB volume was defined as the total volume sent to the laboratory, excluding 35 mL of anticoagulant and the weight of an empty bag (78 g).

TNC was calculated using the formula below (14).

a. TNC count = Absolute number NC/μL × total UCB volume/unit

b. Count of viable nucleated cells = 
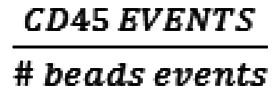
 × 
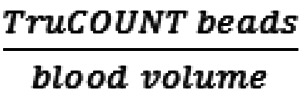
 × Dilution factor  

CD34+ cells were counted with FACSCalibur flow cytometer (Becton Dickinson, Franklin Lakes, NJ, USA). The absolute and total number of NC and CD34^+^ cells was determined with the ISHAGE gating strategy and calculated using the following formulas (15):

a. Absolute CD34^+^ cell count =
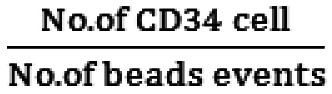
 ×
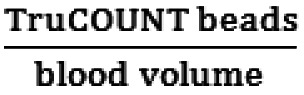
 ×  Dilution factor  

b. Total CD34^+^ cell count = Absolute CD34^+^/ μL × total UCB volume/unit

c. Count of viable CD34^+^ cells = 
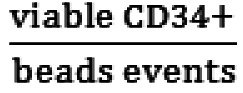
 × 
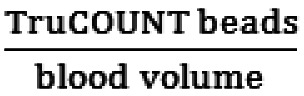
 ×  Dilution factor  

### Variables

Independent variables were maternal age, race, BMI, parity, neonatal sex, gestational age, birth weight, placenta weight, labor duration, and estimated blood loss. Dependent variables were the quality and quantity of HSC.

### Operating definitions

UCB quantity was defined as UCB volume, total TNC count ( × 10^7^/unit), total viable TNC count ( × 10^7^/unit), total CD34^+^ count ( × 10^5^/unit), total viable CD34^+^ count ( × 10^5^/unit), and the percentage of CD34^+^. UCB quality was defined as the percentage of viable TNC and CD34^+^ percentage.

### Statistical analysis

The normality of distribution was assessed with the Z-test using skewness and kurtosis, whereby the absolute value of Z_kurtosis_ and Z_skewness_ lower than -1.96 or higher than 1.96 indicated a distribution deviating from the normal. Categorical variables were compared with the χ^2^ test, whereas continuous variables were compared with the independent *t* test or the Mann-Whitney U-test. Multiple regression analysis was used to identify the factors associated with UCB unit volume and the quantity and quality of TNC and CD34^+^. A *P* < 0.05 was considered statistically significant. The analysis was performed with SPSS, version 21.0 (IBM Corp., Armonk, NY, USA).

## RESULTS

A total of 130 UBC units were procured from 63 mothers with GDM on diet control and 67 healthy mothers. The healthy and GDM group consisted of 94% and 84.1% Malays, respectively. The BMI in the healthy group and GDM group was 25.0 kg/m^2^ and 26.6 kg/m^2^, respectively. The healthy group had a higher percentage of male neonates (57.1% vs 41.8%). The groups had similar mean birth weight, placenta weight, APGAR score, labor duration, and estimated blood loss. The percentage of neonates with macrosomia was comparable (1.5% and 1.6%, respectively), while the percentage of preterm deliveries was three times higher in the GDM group (13.4% vs 4.8%) (Table 1).

**Table 1:** Demographic factors, and maternal and neonatal profile of the healthy and gestational diabetes mellitus (GDM) group

Characteristics	Healthy (n = 67)	GDM (n = 63)	T values or Z values or x^2^ values	*P*
Maternal age (years)				
Mean ± SD	29.1 ± 4.9	30.9 ± 4.3	-2.267^†^	0.025^†^
Median (IQR)	29 (8)	31 (6)		
Minimum and maximum age	18-39	20-43		
Race, n (%)			3.314^§^	0.191^§^
Malay	63 (94)	58 (84.1)		
Chinese	2 (3)	5 (7.9)		
Indian	2 (3)	5 (7.9)		
Parity, n (%)			0.063^§^	0.966^§^
1	18 (26.9)	17 (27)		
2	19 (28.4)	19 (30.2)		
3	20 (29.9)	18 (28.6)		
≥4	10 (14.9)	9 (14.3)		
Maternal body mass index, n (%)			-1.810^†^ 4.839^§^	0.073^†^ 0.089^§^
mean (kg/m^2^)	25.0 ± 4.5	26.6 ± 4.7		
normal	32 (54.2)	17 (33.3)		
overweight	19 (32.2)	24 (47.1)		
obesity	8 (13.6)	10 (19.6)		
Sex of the newborn, n (%)			3.062^§^	0.080^§^
male	36 (57.1)	28 (41.8)		
female	27 (42.9)	39 (58.2)		
Gestational age (weeks), n (%)	38.7 ± 1.5	38.7 ± 1.2	-0.193^†^ 2.914^§^	0.847^†^ 0.088^§^
preterm (<37 weeks)	3 (4.8)	9 (13.4)		
full-term (≥37weeks)	60 (95.2)	58 (86.6)		
Birth weight (grams), mean ± SD	3100 ± 400	3100 ± 400	-0.551^†^	0.583^†^
Macrosomia (>4000 g), n (%)	1 (1.5)	1 (1.6)		
Placenta weight (grams), mean ± SD	577.6 ± 99.0	542.1 ± 112.7	1.901^†^	0.060^†^
APGAR score				
mean ± SD	8.9 ± 0.4	8.9 ± 0.4	0.063^†^	0.950^†^
25th-75th percentile	9-9	9-9	-0.503^‡^	0.615^‡^
Duration of labor (min), mean ± SD	380.9 ± 175.1	386.7 ± 214.2	-0.168†	0.867†
Estimated blood loss, mL				
mean ± SD	240.3 ± 110.2	243.7 ± 88.7	-0.190^†^ -0.718^‡^	0.939^†^ 0.473^‡^
25th-75th percentile	200-250	200-250		

*Abbreviations: SD – standard deviation; IQR – interquartile range; APGAR – appearance, pulse, grimace, activity, and respiration.

†T-test.

‡Mann-Whitney U-test.

§Pearson χ^2^ test.

The mean±SD. UBC volume of 130 UBC units was 57.6 ± 19 mL, total TNC count was 49.8 ± 26 × 10^7^/unit, total viable NC count was 48.5 ± 25.3 × 10^7^/unit, total median CD34+ count (IQR) was 3.6 (7.3), total median viable CD34+ count was 2.4 (4.1), and the percentage of CD34+ was 0.08 (0.13). The mean viable TNC percentage was 96.6 ± 8.2% and viable CD34+ percentage was 71.1 ± 27.4%. The stem cell quantity parameters – UCB volume, total TNCs count, total viable TNCs count, and CD34^+^ percentage were significantly lower in women with GDM compared with the healthy group. Regarding quality parameters, women with GDM had significantly lower viable TNC percentage.

Although the total CD34^+^ and viable CD34^+^ ( × 10^5^/unit) counts were higher in the GDM group, the difference was not significant (Table 2).

**Table 2:** Quantity and quality of umbilical cord blood in the healthy and gestational diabetes mellitus (GDM) group*

Parameters	Healthy group (n = 67)	GDM group(n = 63)	T values or Z values	p for *t* test	p for Mann-Whitney U-test
Quantity of UBC					
UCB volume (mL), mean ± standard deviation	60.88 ± 18.16	54.1 ± 19.4	2.069*	0.041*	
total TNC count ( × 10^7^/unit)¸ mean ± standard deviation	54.8 ± 26.8	44.4 ± 24.1	2.325*	0.022*	
total viable TNC count ( × 10^7^/unit), mean ± standard deviation	53.7 ± 25.5	42.9 ± 24.1	2.482*	0.014*	
total CD34^+^ ( × 10^5^/unit) (25th -75th)	1.4- 9.2	1.7-8.7	-0.813†		0.292 †
total viable CD34^+^ ( × 10^5^/unit) (25th -75th)	0.9- 5.2	1.3-5.4	-1.116†		0.726 †
CD34^+^ (%)(25th -75th)	0.04-0.2	0.1-0.2	2.206†		0.014†
Quality of UBC					
viable TNCs, %	98.4 ± 2.8	94.8 ± 11.1	2.507*	0.015*	
viable CD34^+^, %	71.5 ± 27.2	70.7 ± 27.9	0.165*	0.869*	

*Independent-sample *t* test.

†Mann-Whitney U test.

Multiple regression analysis was used to analyze the factors associated with UCB volume, total TNC count, total viable TNC count, CD34^+^ percentage, and viable TNC percentage among women with GDM. Labor duration, estimated blood loss, sex of the newborn, and placental weight were significantly associated with TNC count. These factors explained 35.6% of the variance in TNC count in the GDM group. Estimated blood loss, sex of the newborn, and placental weight were significantly associated with total viable NC count, explaining 36.6% of the variance (Table 3).

**Table 3:** Factors associated with the quantity and quality of umbilical cord blood in the gestational diabetes mellitus (GDM) group (multiple regression analysis)*

	Umbilical cord blood volume	Total nucleated cells (TNC) count	Total viable NC counts	CD34^+^ (%)	Viable TNC (%)
Constant	-27.97 (0.745)	-55.892 (0.557)	-45.332 (0.631)	-74.498 (0.168)	169.893 (0.009)
Ethnicity (Malay as reference group)	3.799 (0.354)	7.261 (0.112)	7.360 (0.105)	-0.01 (0.997)	1.060 (0.718)
Maternal age	0.462 (0.472)	0.433 (0.541)	0.479 (0.496)	0.100 (0.802)	-0.18 (0.969)
Maternal body mass index	0.299 (0.620)	0.761 (0.253)	0.886 (0.184)	0.254 (0.498)	0.517 (0.235)
Gestational age	-1.119 (0.619)	-1.674 (0.501)	-2.122 (0.391)	1.374 (0.327)	-2.825 (0.086)
Labor duration	0.019 (0.182)	0.031 (0.046)	0.030 (0.052)	0.003 (0.731)	0.003 (0.792)
Estimated blood loss	0.034 (0.213)	0.089 (0.005)	0.092 (0.003)	0.003 (0.866)	0.021 (0.274)
Neonate sex (male as reference group)	5.708 (0.305)	12.322 (0.049)	13.16 (0.035)	-1.77 (0.606	2.854 (0.474)
Birth weight	13.295 (0.120)	8.566 (0.359)	7.637 (0.410)	7.465 (0.159)	-0.498 (0.934)
Placenta weight	0.062 (0.067)	0.080 (0.033)	0.083 (0.026)	0.003 (0.880)	0.019 (0.432)
Adjusted R^2^	0.243	0.356	0.366	0.047	0.120

*Data are presented as unstandardized coefficients B (*P* value).

**Table:** DISCUSSION

In this study, women with GDM had lower stem cell quantity parameters – UCB volume, TNC count, total viable NC count, and CD34+ percentage compared with the healthy group. Regarding quality parameters, they also had lower viable TNC percentage. This is the first study showing that GDM in mothers on a controlled diet negatively affected the quantity and quality of stem cells of UCB.

GDM is commonly associated with various maternal and fetal complications (16). A high glucose level in maternal serum can easily cross the placenta and enter the fetus's bloodstream (17). This directly affects the development and functions of endogenous stem or progenitor cells by stimulating oxidative stress, senescence, and mitochondrial dysfunctions (18,19). GDM negatively affects the proliferation, viability, differentiation, and mitochondrial functions in mesenchymal stem cells obtained from the human umbilical cord (20,21). Our findings accord with those of previous studies (20,21), showing a devastating effect of GDM on the quantity and quality of UBC stem cells regardless of the treatment regimen.

In our study, longer labor duration was associated with a greater TNC count. A possible explanation is that the longer labor duration increases the volume of cord blood due to the stress duration, thus directly affecting the TNC harvested from cord blood. Previous studies also found that labor duration was a crucial determinant of hematopoietic regenerative capacity in UCB (22,23).

Estimated blood loss, sex of the newborn, and placental weight were significantly associated with TNC count and total viable NC count. Consistent with the literature, our research found that male neonates had significantly higher TNC count (24,25). This finding might be explained by a higher mean birth weight (3.2 kg vs 3.0 kg) and higher placental weight (571.7 g vs 502.8 g) of male compared with female neonates. An ideal birth weight for obtaining samples with higher cellularity is around 3.6 kg (26). Another explanation might be the time of clamping and placing of clamps on the cord. A late clamping was associated with a higher hemoglobin concentration in neonates (27,28). However, we did not capture this information at the delivery. In our study, placental weight positively correlated with TNC and total viable NC count, a finding similar to the results of previous reports (29,30). One of the explanations is that placental weight is associated with birth weight (31). In our study, the correlation coefficient between placental weight and birth weight was 0.642 (*P* < 0.001).

A limitation of this study is recruiting a GDM sample from a single tertiary hospital, which makes the results non-generalizable to other populations. In addition, we did not assess the glycemic index throughout the pregnancy; therefore we do not know the outcome of glycemic control of GDM mothers who underwent diet modification program. The neonates born to healthy and those born to GDM mothers had equal birth weights, but the GDM group had a higher number of premature births. Equal neonatal weight could be explained by good glycemic control. However, the higher percentage of preterm deliveries in the GDM group could be explained by hyperglycemia contributing to endothelial dysfunction, which due to lipotoxicity, insulin resistance, and oxidative stress occurring to the fetus, increased the risk of preterm delivery (32-34). Furthermore, this study lacks data on umbilical cord clamping time (which can only affect total blood volume, not concentrations). The difference in placental weights between the groups was very close to the level of statistical probability (especially considering the higher proportion of preterm deliveries in the GDM group), which could also be a source of bias.

This study suggests that the GDM group had much lower quantity and quality of UCB stem cells in contrast to the healthy group. The quantity and quality of UCB are influenced by various maternal and neonatal factors. As processing and cryopreservation of UCB are time-consuming and costly methods, it is essential for the obstetrician to consult the donors carefully. Our results should be taken into consideration when drawing cord blood for unrelated stem cell banking in an obstetric unit to ensure that optimal cord blood samples are obtained and that unnecessary expenses are avoided.
